# Supplementary material for: Hybrid capture RNA-seq defines temporal gene expression in Rickettsia
Source: mSphere. 2026 Apr 30;11(5):e00901-25. doi: 10.1128/msphere.00901-25 (PMC13203960; doi:10.1128/msphere.00901-25)
Supplement: Supplemental material — Fig. S1 to S3 and list of supplemental tables. [file msphere.00901-25-s0001.pdf]

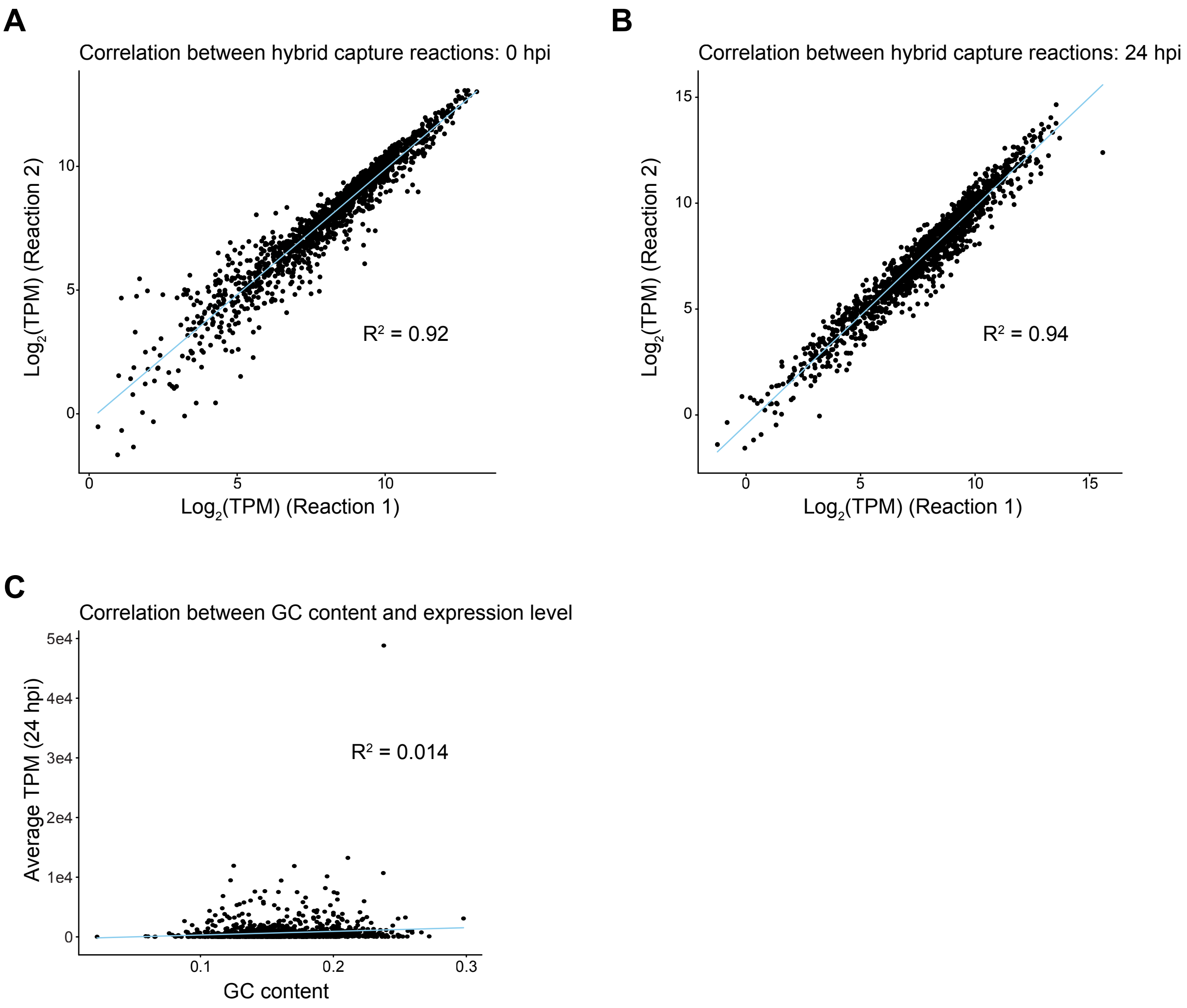

Fig S1 Hybrid capture reactions are reproducible and unaffected by gene GC content. Correlation between hybrid capture reactions of RNA samples collected from biological replicates at 0 hpi (A) and 24 hpi (B). Each reaction contained 6–8 biological replicates per time point and TPM values were calculated as an average of those replicates. (C) Correlation between GC content and gene expression level at 24 hpi.

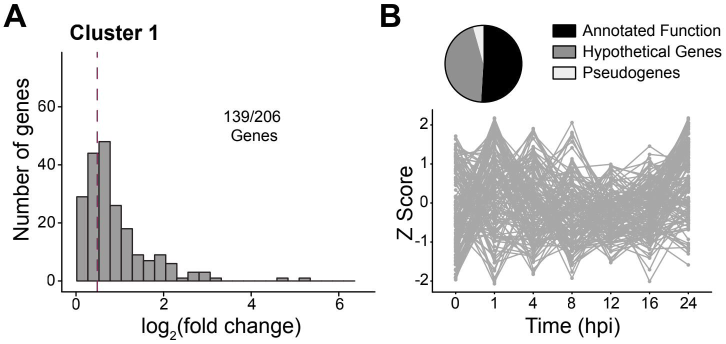

C

GO Term	Description	p value	Fold enrichment
GO:0004803	transposase activity	0.15	4.22
GO:0006629	lipid metabolic process	0.17	2.59
GO:0003677	DNA binding	0.43	1.65
GO:0016757	glycosyltransferase activity	0.58	2.35
GO:0006260	DNA replication	0.99	2.11

Fig S2 Temporal changes and gene content for cluster 1. (A) Histogram log2(FC) for genes in each cluster. Maximum log2(FC) values were calculated as described for Figure 3. (B) Pie charts show the proportion of genes within a cluster annotated as genes of annotated function, hypothetical genes, and pseudogenes. Line graph shows Z score over time of genes in the cluster. (C) Top 5 enriched GO terms within each cluster, reported regardless of statistical significance.

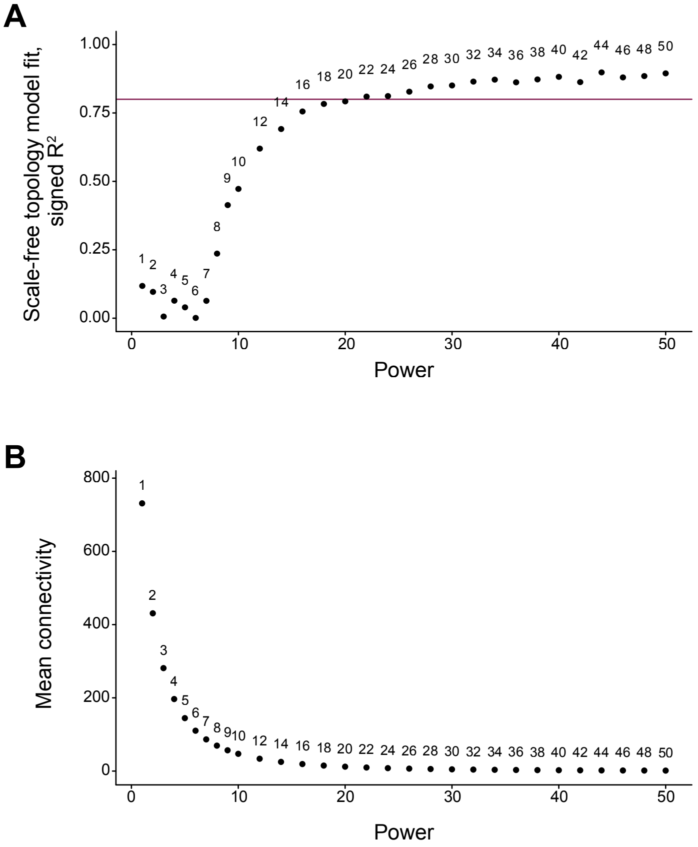

Fig S3 Assessment of soft-thresholding power parameter for WGCNA. (A) Varying values of the soft-thresholding power parameter (β) and the corresponding scale-free topology model or (B) the corresponding mean connectivity values. β was set to 22, which was the smallest power value to fit the scale-free topology model with R2 > 0.8 (red line) that also minimized the mean connectivity value.

Supplemental Tables:

Table S1 Sequencing data for each sample analyzed in this paper, including total read count and reads mapped to the *R. parkeri* genome per sample.

Table S2 DESeq2 analysis of differentially expressed genes comparing 24 and 48 hpi or 24 and 0 hpi. Differentially expressed genes displayed in **Fig. 1D** are denoted by text color.

Table S3 Genes with the highest and lowest average expression across all time course samples and their expression in different conditions from previous works.

Table S4 Cluster and pseudogene, hypothetical, or GO term assignments for all genes in the *R. parkeri* genome included in this work.

Table S5 Putative hub genes identified for each cluster. Clusters not listed did not have associated hub genes. Clusters with more than 20 putative hub genes were limited to the top 20.

Table S6 GO term enrichment data for all clusters as calculated by ClusterProfiler.

Table S7 Operon prediction based on Rockhopper analysis. Genes not listed in this table were predicted to not be in an operon.

Table S8 Correlation, asRNA:mRNA ratio, and associated GO terms for all 629 genes with associated putative asRNA transcripts.
